# The effects of diabetes on attention function: a comparative analysis of children and adolescents with type 1 diabetes and their healthy peers

**DOI:** 10.3389/fendo.2025.1623539

**Published:** 2025-08-12

**Authors:** Paulina Wais, Maia Stanisławska-Kubiak, Elżbieta Niechciał, Katarzyna Anna Majewska, Joanna Wyrwas, Ewa Mojs, Piotr Fichna, Andrzej Kędzia

**Affiliations:** ^1^ Department of Pediatric Diabetes, Auxology and Obesity, Poznan Univeristy of Medical Sciences, Poznan, Poland; ^2^ Department of Clinical Psychology, Poznan University of Medical Sciences, Poznan, Poland; ^3^ Faculty of Health Sciences with the Institute of Maritime and Tropical Medicine, Institute of Nursing and Midwifery, Medical University of Gdansk, Gdansk, Poland

**Keywords:** type 1 diabetes, children, adolescents, neurocognitive function, attention deficit, MOXO-CPT

## Abstract

**Introduction:**

Managing type 1 diabetes (T1D) is complex and requires frequent glucose monitoring, insulin dosing, and lifestyle adjustments to attain appropriate metabolic control. These self-management tasks demand intact cognitive and executive functions, particularly attention. Attention deficits in children and adolescents with T1D have been associated with poor metabolic control and an increased risk of complications. However, research into cognitive performance within this population remains limited. We evaluated attention abilities in children and adolescents with type 1 diabetes compared to healthy controls.

**Materials and Methods:**

The study included 209 children (77 females), comprising 115 with T1D (54 females) and 94 healthy controls (23 females). The mean age of T1D patients was 12.95 years (SD 3.11), with an average disease duration of 5.22 years (SD 3.95). Cognitive functions were assessed using the MOXO Continuous Performance Test (MOXO-CPT), which evaluates attention-related parameters including sustained attention, reaction time, impulsivity, and hyperactivity. The relationship between cognitive performance and clinical parameters, including HbA1c level, treatment methods, glycemic monitoring, and disease duration, was analyzed.

**Results:**

Children with T1D demonstrated significantly lower sustained attention scores, slower reaction times, and worse hyperactivity levels than controls. Impulsivity did not differ significantly. Patients with HbA1c levels greater than 8% showed noticeably poorer attention performance. Gender, disease duration, treatment method, and type of glycemic monitoring were not associated with attention outcomes.

**Conclusions:**

Children and adolescents with T1D exhibit worse neurocognitive performance, particularly in attention, compared to healthy peers. Poor metabolic control is linked to attention deficits. Routine cognitive screening of children and adolescents with T1D may enhance disease management and highlight the need for additional support in therapeutic tasks.

## 1 Introduction

Type 1 diabetes (T1D) is one of the most common chronic diseases affecting the pediatric population, and its incidence is still rising worldwide. According to the 11th edition of the International Diabetes Federation (IDF), the number of children and adolescents younger than 20 years living with T1D reached more than 1.9 million in 2024 globally, with almost 219,000 new cases diagnosed yearly. Moreover, the age of diagnosis for T1D is also decreasing (1). This upward trend is observed globally, with countries such as Poland, Turkey, Kuwait, Qatar, and Canada are reporting significant increases (2–6).

T1D is caused by immune-mediated destruction of insulin-producing beta cells in the pancreas, which results in insulin deficiency and chronic hyperglycemia. Its management is complex and demands rigorous daily self-care, including insulin administration, diet regulation, physical activity, and psychosocial stressors such as puberty or illness (7, 8).

Over time, individuals with T1D might experience various acute (hypoglycemia, diabetic ketoacidosis) and chronic complications (microangiopathies and macroangiopathies) that lead to impaired function of many systems and organs, including those affecting the central nervous system structure and function (9, 10).

Cognitive functions include attention, memory, language, and executive abilities responsible for planning and self-regulation. Their development follows a sequential trajectory throughout childhood and adolescence, guided by neurobiological maturation and environmental input (11). In the context of chronic illnesses such as T1D, this delicate trajectory may be disrupted. Adolescents with T1D are especially vulnerable, as metabolic instability during critical periods of brain development may lead to lasting neurocognitive deficits. Early-onset T1D, particularly before age five, is associated with poorer outcomes in intelligence, academic achievement, and executive functioning, as shown in longitudinal studies (12–15).

Chronic dysglycemia during these critical developmental windows may impair neural plasticity, neurogenesis, and synaptic functioning through mechanisms involving oxidative stress, neuroinflammation, and microvascular damage (16–18).

Beyond its impact on metabolic outcomes, impaired cognitive functioning in T1D can also affect emotional regulation, social integration, and academic achievement (19, 20). Given these complexities, a comprehensive neuropsychological evaluation should be considered a key component of T1D management in pediatric populations. Standardized cognitive assessments, such as computerized continuous performance tests (e.g., MOXO-CPT), offer objective and reliable measures of attentional control, impulsivity, and processing speed. These tools enable clinicians to identify cognitive difficulties early and tailor interventions to support both metabolic and psychosocial outcomes (21–24).

Despite numerous studies on the co-occurrence of T1D and cognitive function disorders in children and adolescents, there are currently no clear guidelines regarding therapeutic approaches for this patient group. Additionally, there is no established requirement in the guidelines for the treatment of T1D in children and adolescents for conducting screening tests for cognitive function disorders in these patients.

This study aims to conduct a comparative analysis of attention parameters in children and adolescents with T1D and their healthy peers using the computerized MOXO Continuous Performance test (MOXO-CPT).

## 2 Materials and methods

### 2.1 Participants’ demographic characteristics and group stratification

#### 2.1.1 Participants’ demographic characteristics

The study cohort comprised 209 children (77 females), including 115 (54 females) with T1D (55.0%) and 94 (23 females) healthy children forming the control group (45.0%). Children with T1D were recruited from the Department of Pediatric Diabetology, Auxology, and Obesity in Poznan, Poland. Participants’ age ranged from 6 to 18 years (|M| = 12.95; SD 3.11), with disease duration ranging from 1 to 15 years (|M|= 5.22; SD 3.95). The mean HbA1c was 7,51% (SD 1,53). Fifty-two patients with T1D were treated with continuous subcutaneous insulin infusion (CSII), and sixty-three received multiple daily injections (MDI). Thirty-eight patients with T1D monitored their blood glucose levels using continuous glucose monitoring (CGM), which included flash glucose monitoring (FGM), while the remaining participants relied on self-monitoring of blood glucose (SMBG) using a standard blood glucose meter. The healthy children were between 6 and 18 years (M = 13.03; SD 3.43). The demographic similarity of participants, drawn from comparable urban public schools within the same region and with similar access to healthcare services, likely reduced variability related to socioeconomic background, thereby strengthening the validity of between-group comparisons.

#### 2.1.2 Participants’ group stratification

To enable more detailed analysis of attentional performance at MOXO-CPT test, participants with T1D were stratified into subgroups based on clinically relevant variables: gender (girls and boys), age (6–12 years, 13–15 years, and (16–18 years), level of glycemic control (HbA1c <7%, HbA1c >8%), diabetes duration (≤5 years, >5–10 years, and >10 years), glucose monitoring (CGM, SMBG), type of treatment (CSII, MDI).

The division into age groups (6–12 years, 13–15 years, and 16–18 years) was selected due to developmental differences and stages of maturation that may influence cognitive functions, including attention. Middle childhood (6–12) involves high brain plasticity and rapid growth; early adolescence (13–15) features neural reorganization and increased autonomy; late adolescence (16–18) sees advanced reasoning but still developing decision-making. This grouping aligns with key brain development stages and facilitates meaningful interpretation of findings related to T1D management (25–27).

To evaluate the relationship between glycemic control and cognitive performance, a subgroup analysis was conducted based on HbA1c levels, utilizing clinically validated thresholds (28). Participants were categorized into two groups: those with HbA1c <7%, indicative of good glycemic control, and those with HbA1c >8%, reflecting poor metabolic regulation. No participants in the study group obtained a score between 7% and 8%.

Diabetes duration was divided into three intervals: ≤5 years, >5–10 years, and >10 years, reflecting progressive stages of cumulative metabolic burden. Duration T1D under 5 years likely involves minimal risk of cognitive decline due to limited exposure. The 5–10-year range may see early cognitive effects from cumulative glycemic variability and metabolic stress, especially during school years. Beyond 10 years, prolonged metabolic fluctuations increase the risk of early impairments in attention and processing speed, making this group more vulnerable to long-term neurocognitive effects. These stratification criteria were informed by prior pediatric research (14, 15). This stratification allowed for the assessment of how disease-related factors may differentially affect attentional performance.

Participants diagnosed with psychological or psychiatric disorders, neurological conditions, or severe diseases requiring burdensome treatment, apart from diabetes, were excluded from the study. Informed consent was required from the parents of all participants and directly from participants who were 16 years of age or older. The presented research is in accordance with the World Medical Association Declaration of Helsinki - Ethical Principles for Medical Research Involving Human Subjects World Medical Association Declaration of Helsinki - Ethical Principles for Medical Research Involving Human Subjects (29). The study was approved by the Bioethics Committee at the Poznan University of Medical Sciences (No 1140/19).

### 2.2 Monitoring of glycemia and additional tests

An extensive medical history regarding glycemic self-monitoring was collected from patients with T1D. Readings were obtained from CGM systems, as well as from glucose meters. HbA1c level was measured for each patient during the visit. Five minutes before the test, capillary blood glucose levels were measured using a glucometer. Children and adolescents presenting with capillary blood glucose levels below 70 mg/dL (hypoglycemia) or above 250 mg/dL (hyperglycemia), as measured five minutes before testing, were excluded from participation. Glycemic variability was not continuously monitored during the MOXO-CPT performance. Instead, a pre-test blood glucose measurement was used as the sole eligibility criterion to ensure metabolic stability at the beginning of the assessment. This strategy was adopted due to the relatively short duration of the test (15–18 min.).

### 2.3 Continuous performance test

Cognitive performance, with a primary focus on attentional functioning, was evaluated using the MOXO Continuous Performance Test (MOXO-CPT), a computerized tool designed to assess attention-related difficulties in children and adolescents (22, 30). The pediatric version, administered to participants aged 6–12 years, lasted approximately 15 minutes, while the adolescent version (for those aged 13 years and older) lasted 18.5 minutes. Before testing, each participant received standardized instructions and completed a brief practice trial to ensure comprehension of task demands. The MOXO-CPT comprises eight sequential phases, each varying in distractors’ type, intensity, and modality to simulate real-world attentional challenges. Participants are required to respond to a predefined visual target stimulus by pressing the spacebar as quickly and accurately as possible whenever the target reappears. Throughout the task, non-target stimuli - visual, auditory, or combined audiovisual distractors- are introduced to evaluate the participant’s capacity to maintain focus and suppress irrelevant information. The test dynamically modulates distractor complexity to provide a real-world applicable measure of attentional control (30).

Outcome measures include four core indices: sustained attention, impulsivity, hyperactivity, and reaction time. Sustained attention reflects consistently detecting and responding to relevant stimuli over time. Impulsivity captures premature or inappropriate responses, while hyperactivity measures excessive or unnecessary motor activity during the task. Reaction time reflects the speed and efficiency of stimulus processing and response execution. Results were interpreted relative to age- and sex-adjusted normative data. For each indicator, group norms are used to calculate a Z-score, which is then categorized into one of four levels: Z ≥ 0 indicates a good result (within the high norm); –0.825 ≤ Z < 0 represents a standard result; –1.65 ≤ Z < –0.825 is interpreted as a low result (within the low norm); and Z < –1.65 indicates functional difficulties. In addition, the severity levels of deviation are calculated only for indicators that fall below the normative range (Z < -1.65). Higher scores in the assessed domains indicate better cognitive performance.

**Figure 1:**
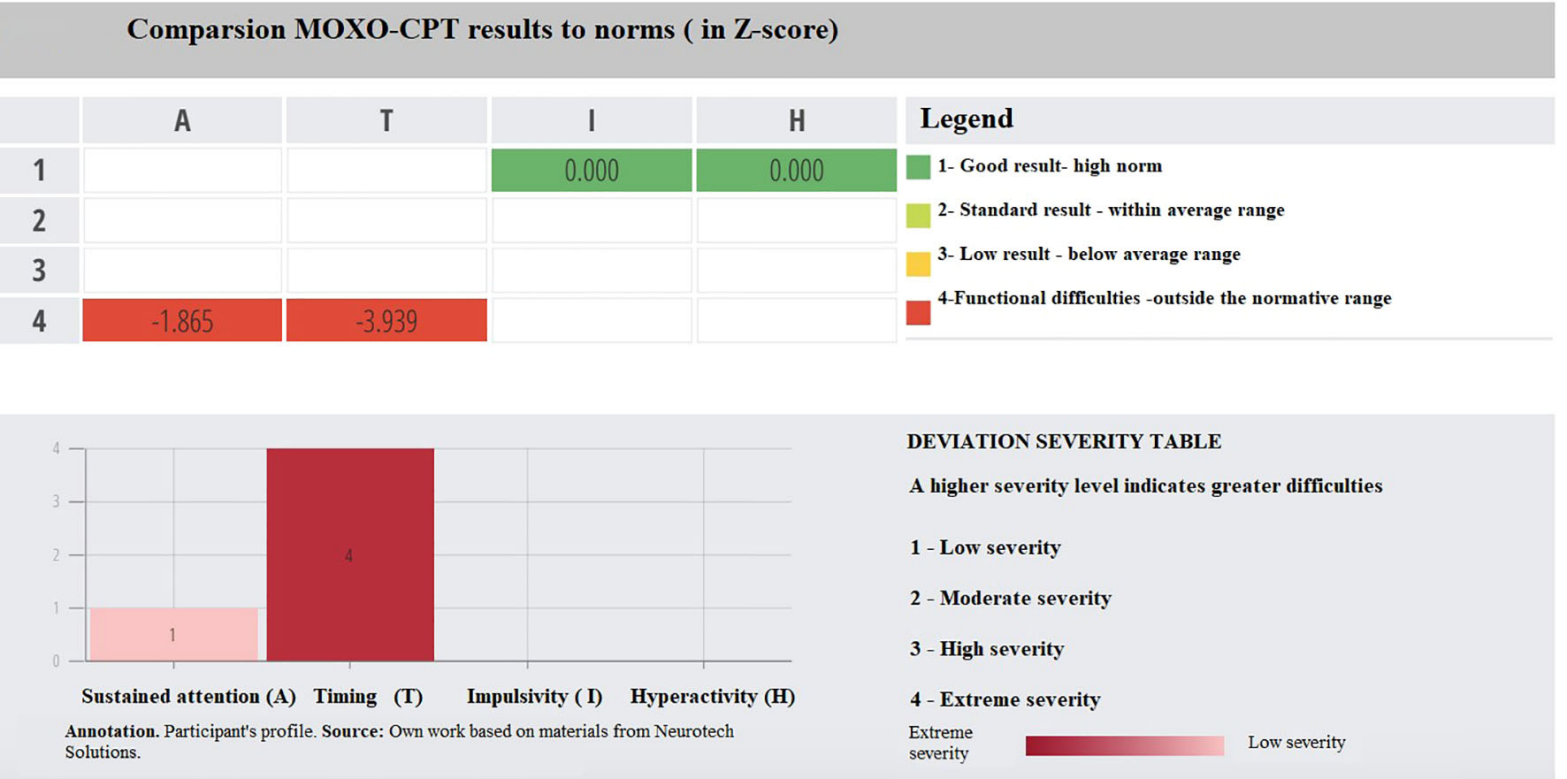
Comparsion MOXO-CPT results to norms (in Z-score).

**Figure 2:**
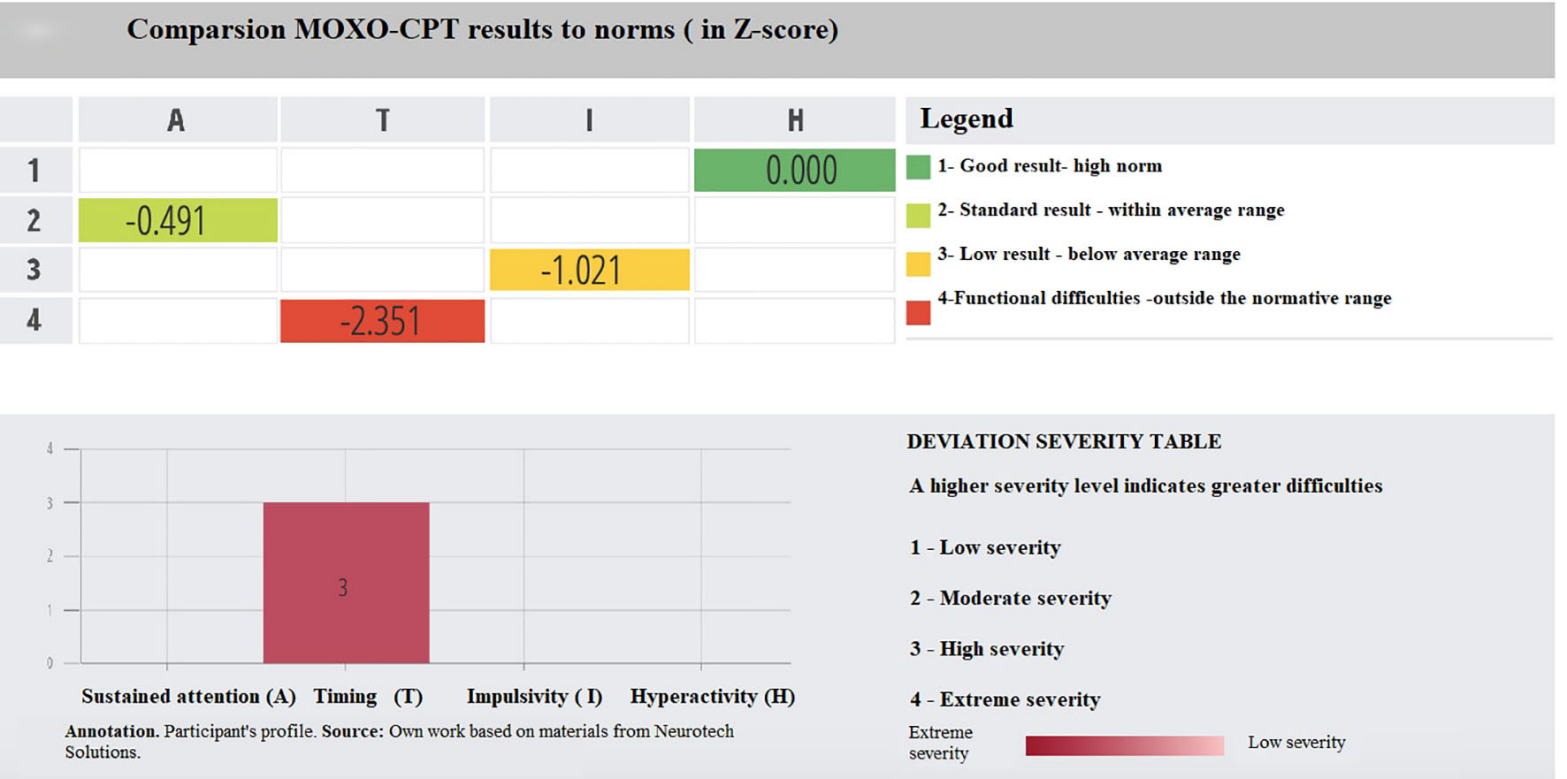
Comparsion MOXO-CPT results to norms (in Z-score).

To enhance interpretability, **Figures 1** and **2** present MOXO-CPT profiles of individual participants. The sample patient profile shown in **Figure 1** represents an individual who obtained scores outside the normative range in the domains of sustained attention and reaction time, while achieving high normative scores in impulsivity and hyperactivity. For scores at level 4 (indicating functional difficulties), the severity of impairment is further categorized from low to extreme. In this case, the deviation in reaction time was classified as having extreme severity, while the deviation in sustained attention was classified as having low severity. **Figure 2** displays the MOXO-CPT profile of a participant who achieved a standard result in the sustained attention domain. In the reaction time domain, functional difficulties were observed, with the deviation rated as high severity. The impulsivity score was within the below average range, while the hyperactivity domain showed a result within the high normative range.

In our study, all participants completed the test under standardized conditions. After the assessment, feedback regarding the test results was provided to each patient and their caregiver.

### 2.4 Statistical analysis

Statistical analyses were conducted using IBM SPSS Statistics 30. A p-value of ≤ 0.05 was considered statistically significant.

A series of statistical tests were used to verify the study hypotheses and assess differences between the study groups. The chi-square test was applied to examine relationships between categorical variables. To compare groups on multiple continuous outcomes derived from the MOXO-CPT, univariate (1-way MANOVA) and bivariate/multivariate analyses of variance (2- way MANOVA) were performed.

Additionally, due to the categorical nature of the MOXO output scores (i.e., severity levels), chi-square tests of independence were conducted separately for each MOXO domain to examine distribution differences by gender. Cramér’s V coefficients were also calculated to assess the strength of associations.

Interpretation of the effects applied to the criteria used by Cohen (31), which spread for small, medium, and large effects, and the practical application of the results. The normality of the distribution was assessed using skewness and kurtosis values. In line with the guidelines proposed by George and Mallery (32), values within the range of −2 to +2 were considered indicative of an approximately normal distribution. All analyzed variables fell within this range and were therefore treated as normally distributed for the purposes of parametric analysis. Additional information regarding the analysis of variance (ANOVA) is based on the assumption of normality of the distribution, according to the research results by Schmider et al. (33), indicated that ANOVA is indeed available for departures from normality.

## 3 Results

To estimate the difference between the T1D group and the control group, a two-factor multivariate analysis of variance (2-way MANOVA) was conducted. The first independent factor was group membership (**Table 1**), while the second independent factor was the division based on age (**Table 2**).

**Table 1:** Comparison of MOXO-CPT test results in children with T1D and healthy children.

MOXO-CPT	Group name	*n*	*Mean*	*SE*	*F* (1,203)	*P*	η^2^
Sustained attention	Control	94	0,375	0,225	10,95	0,001	0,05
	T1D	115	-0,628	0,203			
Timing	Control	94	-0,275	0,177	8,12	0,005	0,04
	T1D	115	-0,956	0,160			
Hyperactivity	Control	94	0,852	0,203	5,84	0,017	0,03
	T1D	115	0,192	0,183			
Impulsivity	Control	94	-0,141	0,254	0,02	0,885	<0,01
	T1D	115	-1,91	0,229			

*M*, mean; *SE*, standard error; *p*, statistical significance; η², effect strength indicator; F, value of the test statistic; T1D, children and adolescents with diabetes type 1; *n*, number of participants.

**Table 2:** MOXO scale scores vary depending on the interaction of age and study group.

MOXO-CPT	Group	6–12 y	13–15 y	16–18 y	ME – Control vs T1D		ME – age		IE - age x (control vs T1D)	
	*n* =94/115	*n*=39/51	*n* =23/32	*n* =32/32						
					η^2^	*P*	η^2^	*P*	η^2^	*P*
Sustained attention	Control	0,35 (1,09)	0,61 (1,25)	0,16 (1,05)	0,05	**0,001**	<0,01	0,922	0,01	0,312
	T1D	-0,53 (2,22)	-1,05 (3,69)	-0,31 (2,09)						
Timing	Control	0,03 (1,44)	-0,33 (1,05)	-0,53 (2,05)	0,04	**0,005**	0,01	0,318	0,00	0,842
	T1D	-0,81 (1,79)	-1,01 (1,73)	-1,05 (1,66)						
Hyper-reactivity	Control	0,98 (1,08)	0,72 (0,87)	0,85 (0,70)	0,03	**0,017**	0,02	0,122	0,02	0,131
	T1D	-0,07 (2,22)	-0,32 (3,64)	0,97 (0,69)						
Impulsivity	Control	-0,01 (1,20)	-1,87 (1,85)	-1,54 (1,69)	<0,01	0,885	0,11	**<0,001**	0,03	0,054
	T1D	-0,43 (2,06)	-2,78 (4,36)	-0,37 (2,21)						

*M*, mean; *p*, statistical significance; η², effect strength indicator; ME, main effect; IE, interaction effect; *n*, (Control/T1D).

The bolded values indicate statistically significant result ( p<0.05).

**Figure 3:**
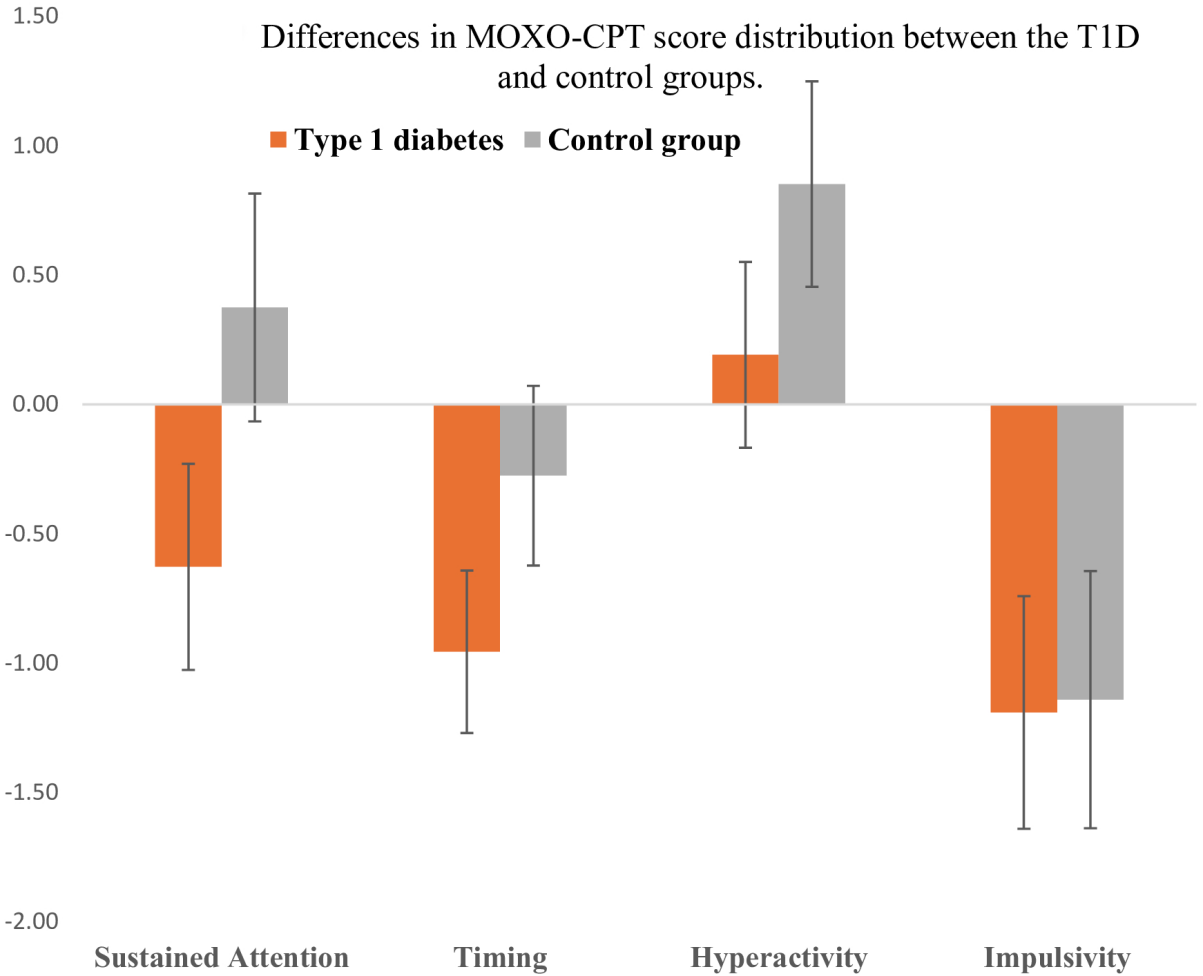
Differences in MOXO-CPT score distribution between the T1D and control groups. Error bars are 95% confidence intervals of the mean results.

Analysis of variance (2-way MANOVA) (**Table 1**) revealed significant main effects for the study group (T1D vs. control group) in three variables: sustained attention *F*(1,203) = 10.95, p=0.001, η²=0.05, reaction time *F*(1,203) = 8.12, p = 0.005, η² = 0.04, and hyperactivity *F*(1,203) = 5.84, p = 0.017, η² = 0.03. No significant effect was observed for impulsivity *F*(1,203) =0.02, p = 0.885, η² < 0.01. Mean values for each group indicate that individuals with T1D scored significantly lower in sustained attention (-0.63, SE=0.20) compared to the control group (0.38, SE=0.23). Regarding reaction time, individuals with T1D (-0.96, SE=0.16) exhibited slower reaction times than healthy individuals (-0.28, SE=0.18). A similar pattern was observed for hyperactivity, where individuals with T1D displayed a worse score of the hyperactivity variable (M = 0.19, SE = 0.18) than the control group (M = 0.85, SE = 0.20). These between-group differences in MOXO-CPT performance are illustrated in **Figure 3**, which presents the variation in test scores across the main cognitive domains.

In the next stage, a two-factor multivariate analysis of variance (2-way MANOVA) was performed to estimate the differences in the studied areas of the MOXO-CPT test between the T1D group and the control group, depending on age. The study participants were divided by age: 6–12 years vs. 13–15 years vs. 16–18 years. The data analysis is presented in **Table 2**. The analysis of age revealed a significant main effect on impulsivity. However, no significant differences were found in the other variables - sustained attention, reaction time, and hyperactivity (p > 0.05). The mean values indicate that impulsivity varied depending on age, with the highest negative level of impulsivity observed in the 13–15 age group. In contrast, the youngest (6–12 years) and oldest (16–18 years) groups showed higher, more positive values. Multiple comparisons revealed that the 13–15 age group differed significantly from the 6–12 age group (p < 0.001) and significantly from the 16–18 age group (p = 0.007).

**Figure 4:**
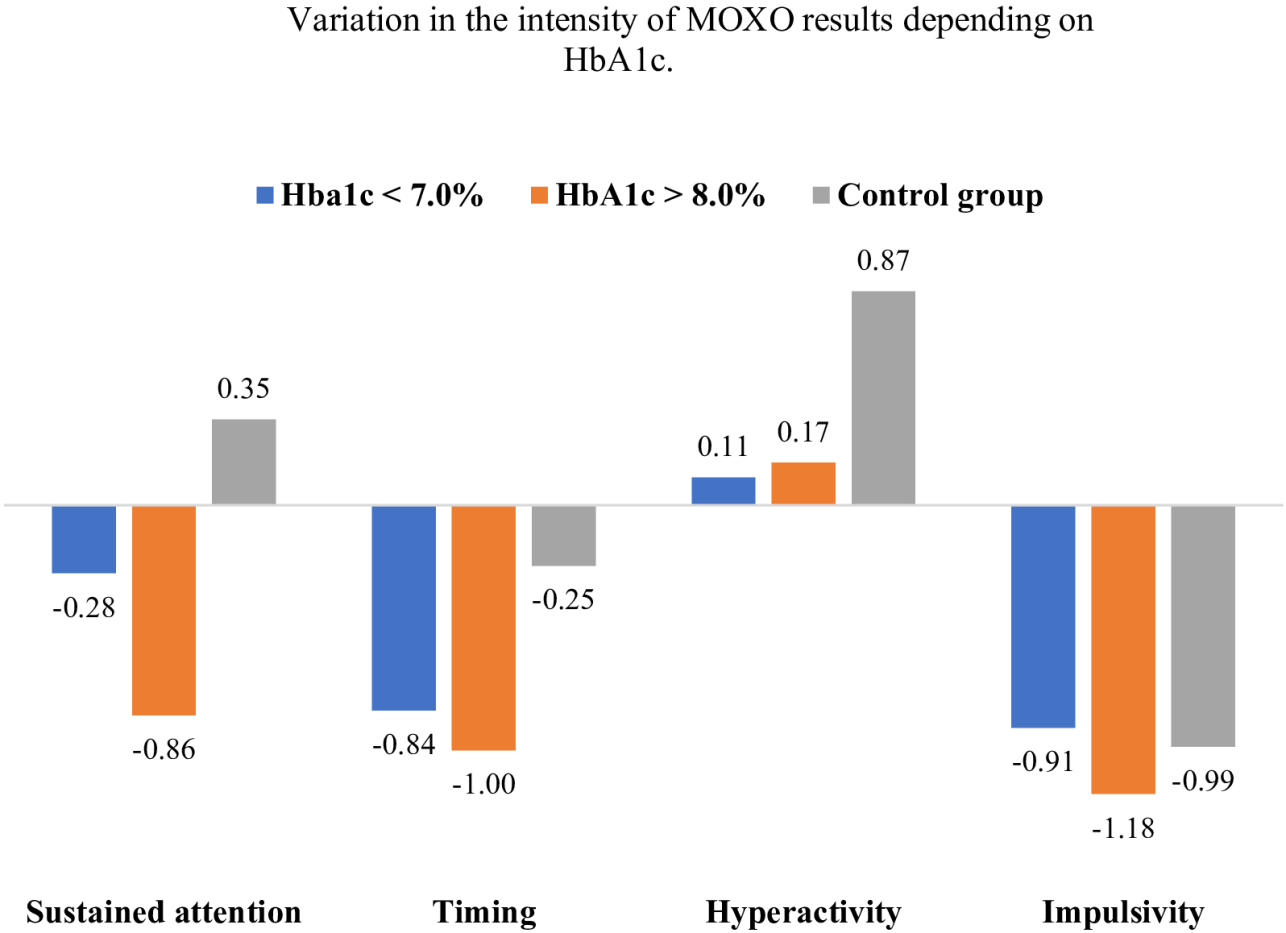
Variation in the intensity of MOXO results depending on HbA1c. Error bars are 95% confidence intervals of the mean results.

In the analysis of MOXO-CPT performance based on the predefined HbA1c stratification, 49 participants had HbA1c levels below 7%, and 66 participants had levels exceeding 8%. Notably, no individuals with T1D in the study sample had HbA1c values within the intermediate range of 7% to 8%, allowing for a distinct comparison between well-controlled and poorly controlled diabetes groups. The analysis of variance (**Figure 4**) revealed significant main effects for three variables depending on HbA1c values: sustained attention *F*(2,206) = 6.42, p = 0.002, η² = 0.06, reaction time *F*(2,206) = 4.45, p = 0.013, η² = 0.04, and hyperactivity *F*(2,206) = 3.61, p = 0.029, η² = 0.03. The η² values indicate a small effect size in these cases. No significant main effect was observed for impulsivity *F*(2,206) = 0.18, p = 0.836, η² = 0.002.

Regarding sustained attention, individuals with HbA1c > 8.0% scored significantly lower than the control group (difference = -1.21, SE = 0.34, p = 0.001). The difference between the HbA1c < 7.0% and control groups was not significant (difference = -0.63, SE = 0.37, p = 0.23). The difference between the HbA1c > 8.0% and HbA1c < 7.0% groups was not significant (difference= -0.58, SE = 0.40, p = 0.440). For reaction time, individuals with HbA1c >8.0% scored significantly lower than the control group (difference = -0.75, SE = 0.27, p = 0.017), whereas the difference between the HbA1c > 8.0% and HbA1c < 7.0% groups was not significant (difference = -0.16, SE = 0.32, p = 1.000). The difference between the HbA1c < 7.0% and control groups was not significant (difference = -0.59, SE = 0.3, p=0.14). In the ANOVA, a significant main effect was observed for the variable hyperactivity (F(2,206) = 3.61, p = 0.029, η² = 0.03), indicating the presence of overall differences among the analyzed groups. However, subsequent *post hoc* comparisons did not reveal statistically significant pairwise differences. Individuals with T1D had numerically lower hyperactivity scores compared to the control group (HbA1c > 8.0% vs. control: difference = -0.70, SE = 0.31, p = 0.079; HbA1c < 7% vs. control: difference = -0.76, SE = 0.34, p = 0.083). No significant differences in impulsivity were observed among the analyzed subgroups.

A two-factor, multivariate analysis of variance was also performed to assess the effect of gender on the results of the studied parameters of the MOXO test, comparing the group of patients with T1D with the control group. Analyzing the results presented in **Table 3**, no statistically significant differences were found based on gender or gender by group interaction.

**Table 3:** Variation in MOXO scale scores depending on the interaction of gender and study group.

MOXO-CPT	Gender	Control *n* = (71/23)	T1D *n* = (61/54)	ME gender		ME (control vs T1D)		IE - Gender x (control vs T1D)	
				η^2^	*P*	η^2^	*p*	η^2^	*P*
Sustained attention	Boys	0,23 (1,12)	-0,80 (3,01)	0,01	0,167	0,05	**0,001**	<0,01	0,888
	Girls	0,72 (1,06)	-0,40 (2,24)						
Timing	Boys	-0,32 (1,58)	-1,15 (1,70)	0,01	0,141	0,04	**0,004**	<0,01	0,720
	Girls	-0,03 (1,69)	-0,68 (1,73)						
Hyperactivity	Boys	0,90 (0,72)	-0,07 (2,76)	<0,01	0,572	0,03	**0,022**	<0,01	0,322
	Girls	0,78 (1,35)	0,39 (2,12)						
Impulsivity	Boys	-1,13 (1,75)	-1,24 (2,59)	0,01	0,230	0,00	0,591	<0,01	0,810
	Girls	-0,56 (1,70)	-0,86 (3,57)						

M, mean; p – statistical significance; η² - effect strength indicator; ME, main effect; IE, interaction effect, F- the value of the test statistic, T1D- children and adolescents with diabetes type 1; *n*=(boys/girls).

The bolded values indicate statistically significant result ( p<0.05).

The chi-square tests of independence and Cramér’s *V* coefficients, summarized in **Table 4**, revealed no statistically significant differences in the distribution of performance categories between boys and girls across any of the MOXO domains.

**Table 4:** Sex-related variation in MOXO-CPT outcomes.

MOXO-CPT	Severity levels of parameters	Boys		Girls		χ^2^(3)	*P*	*V*
		*n*	%	*N*	%			
Sustained attention	*Impaired performance*	17	13,0%	12	15,6%	5,80	0,122	0,17
	*Weak result*	10	6,9%	1	1,3%			
	*Standard score*	21	16,0%	7	9,1%			
	*Good result*	84	64,1%	57	74,0%			
Timing	*Impaired performance*	32	23,8%	19	24,7%	0,99	0,804	0,07
	*Weak result*	21	15,4%	9	11,7%			
	*Standard score*	32	24,6%	17	22,1%			
	*Good result*	47	36,2%	32	41,6%			
Hyperactivity	*Impaired performance*	13	9,8%	5	6,5%	1,26	0,738	0,08
	*Weak result*	2	1,5%	2	2,6%			
	*Standard score*	9	6,8%	7	9,1%			
	*Good result*	108	81,8%	63	81,8%			
Impulsivity	*Impaired performance*	41	31,1%	22	28,6%	5,37	0,147	0,16
	*Weak result*	24	18,2%	6	7,8%			
	*Standard score*	20	15,2%	13	16,9%			
	*Good result*	47	35,6%	36	46,8%			

*n*, number of participants; χ², Chi-square analysis; *p*, statistical significance; *V*, effect size measure.

In the subsequent phase of the analysis, the effects of the treatment method and glucose monitoring system on cognitive function parameters were examined. The analyses were conducted using a one-way analysis of variance (ANOVA). No statistically significant differences were found between the CSII and MDI groups across any of the assessed MOXO-CPT parameters (p = 1.000). *Post hoc* analyses revealed several group-specific effects. In the domain of sustained attention, a statistically significant difference was observed between the CSII group and the control group (p = 0.007), with the CSII group obtaining significantly lower scores. In the reaction time domain, the MDI group showed significantly longer reaction times compared to controls (p = 0.045). A non-significant difference was also observed between the CSII and control groups in this domain (p = 0.066). Regarding hyperactivity, the MDI group demonstrated significantly higher levels than the control group (p = 0.024). No significant differences were found between the CSII and control groups in this domain (p = 0.298). In the domain of impulsivity, no statistically significant differences were observed among the groups (p > 0.05).

Similarly, no significant differences were detected between patients using CGM and those using SMBG (p = 1.000). However, *post hoc* analysis revealed that patients using the CGM system had significantly longer reaction times than the control group (p = 0.017), suggesting reduced attentional efficiency. The SMBG group also showed significantly higher hyperactivity scores compared to controls (p = 0.042).

The results of the analysis of the duration of T1D, considering the division into ≤5 years, >5–10 years, and >10 years, did not show any significant differences between the groups in terms of the tested MOXO test parameters.

## 4 Discussion

The present study contributes to the growing body of literature exploring the neurocognitive sequelae of T1D in the pediatric population. Our findings indicate significant differences in sustained attention, reaction time, and hyperactivity among children and adolescents with T1D compared to healthy controls. Children with poorly controlled T1D (HbA1c > 8%) showed significantly lower attention scores compared to healthy controls, suggesting an adverse effect of chronic hyperglycemia. No significant difference was observed between well- and poorly controlled patients, possibly indicating a threshold beyond which cognitive deficits become more evident. The lack of mid-range HbA1c values limits interpretation across the full glycemic spectrum. Poorer performance in four attention parameters among patients with T1D, compared to the control group, was also confirmed in the study conducted by Lancrei et al. (2022), particularly among individuals with early-onset T1D, longer disease duration, and poorer glycemic control (23).

A recent study by Kar et al. (2023) also used the MOXO-CPT to evaluate neurocognitive functioning in children with T1D and found increased impulsivity compared to healthy controls (34). While their findings regarding impulsivity differ from ours, both studies highlight the presence of attention-related challenges in youth with T1D. Notably, Kar et al. also reported a significant association between poorer metabolic control and greater cognitive impairment, which aligns with our findings. In our larger sample, we observed significant differences in MOXO domains other than impulsivity. This suggests that attention may be affected in various ways in children with T1D, even if the specific types of deficits differ across studies.

It is worth noting that participants with T1D and those in the control group in our study obtained generally high scores in the hyperactivity domain. However, a statistically significant difference was observed between the groups, with patients with T1D showing lower performance in this domain. This result may be interpreted either as an adaptive advantage, possibly stemming from the self-discipline and attentional control required in daily diabetes management, or as a manifestation of fatigue or behavioral withdrawal resulting from the chronic burden of illness (35). This result underscores the complex nature of neurocognitive functioning in individuals with T1D and highlights the need for further investigation.

Although cognitive impairments were more pronounced among participants with HbA1c levels exceeding 8%, broader patterns in the data suggest that the chronic nature of T1D itself, rather than poor metabolic control alone, may be a central contributor to these cognitive vulnerabilities.

This observation implies that cognitive challenges in individuals with T1D are not exclusively attributable to glycemic control but may also arise from long-term neurodevelopmental consequences associated with living with a chronic illness. These include repeated exposure to glycemic variability, ongoing psychosocial stress, and the sustained cognitive demands of daily diabetes management.

Therefore, while suboptimal glycemic control may exacerbate neurocognitive difficulties, our findings support the view that T1D per se constitutes a fundamental risk factor for attentional dysfunction (36).

Our findings revealed no significant sex-related differences in MOXO-CPT performance across all assessed domains, including attention, timing, hyperactivity, and impulsivity, suggesting that sex did not substantially influence attentional performance in this sample. This observation is consistent with previous studies (23). These results suggest that attentional deficits associated with type 1 diabetes are not moderated by sex, thereby reinforcing the utility of the MOXO-CPT as a reliable tool for detecting diabetes-related cognitive difficulties in both girls and boys. Given that the test is standardized for both age and sex, the absence of sex differences further supports the robustness of MOXO-CPT scores and their clinical interpretability across diverse pediatric populations.

The MOXO-CPT, originally developed as an objective and standardized diagnostic tool for attention-related disorders such as ADHD, has increasingly been employed in broader pediatric neuropsychological assessments, including those involving chronic somatic conditions, thereby reinforcing its clinical utility (21–24). Previous studies using behavioral questionnaires, such as the Conners’ Rating Scales, which primarily capture subjective perceptions from parents or teachers, have similarly reported impairments in sustained attention and reaction time among pediatric T1D cohorts (37).

Ibrahim et al. (2023) reported significantly lower scores on the Modified Mini-Mental State Examination (MMMS) and the Pediatric Symptoms Checklist (PSC) among children with T1D compared to healthy controls. These results suggest deficits in attention, memory, and psychosocial functioning (38). Furthermore, meta-analyses utilizing standardized neurocognitive tools, such as the Attentional Network Test (ANT), have consistently demonstrated reduced processing speed and sustained attention in youth with T1D (39). The convergence of findings across diverse methodologies, both objective and subjective, substantiates the clinical relevance of attentional dysfunction in children with T1D and strengthens the validity of the MOXO-CPT as a reliable neuropsychological assessment tool in this population. The MOXO-CPT is relatively quick to administer (~15 minutes), requires minimal professional training, and is engaging for children. These features make it particularly suitable for use in outpatient pediatric care settings—including endocrinology and diabetes clinics—by trained nurses or allied health professionals.

When cognitive difficulties are identified, the results can inform a dual-intervention approach: optimizing diabetes management to improve self-care and treatment adherence, while also facilitating timely referrals to psychological services for broader cognitive and emotional support.

### 4.1 Attention and executive function in T1D: neurodevelopmental considerations

Attentional processes serve as a foundational component of executive functioning, which in turn underpins an individual’s capacity for planning, problem-solving, and managing complex, goal-directed behaviors. In the context of T1D, sustained attention and cognitive flexibility are critical for effective self-management behaviors such as blood glucose monitoring, insulin administration, dietary regulation, and interpretation of real-time glycemic data. Deficits in these cognitive domains may compromise disease management, increasing the risk of acute complications (hypoglycemia, hyperglycemia) and contributing to long-term sequelae (15).

### 4.2 Age and impulsivity: a developmental perspective

An age-related effect was observed with respect to impulsivity, which was more pronounced in early adolescence (ages 13–15). This finding is consistent with developmental neurobiology, wherein subcortical structures responsible for reward processing (e.g., the limbic system) mature earlier than the prefrontal cortex, which governs inhibitory control and executive functioning (27, 40, 41). The asynchronous development of these systems may predispose adolescents to heightened impulsivity, a phenomenon further exacerbated by the metabolic instability characteristic of puberty. The pubertal period is associated with increased secretion of growth hormone and sex steroids, contributing to greater glycemic variability (42).

This metabolic instability, combined with the normative increase in psychosocial stress and the transfer of diabetes self-management responsibilities to the adolescent, may impose additional cognitive burdens (43, 44).

### 4.3 Technological advances in glycemic monitoring and diabetes treatment: a double-edged sword?

While the introduction of advanced technologies, such as CGM or CSII, has revolutionized diabetes management by improving glycemic control and reducing the frequency of severe hypoglycemic events, our findings suggest potential unintended consequences on cognitive functioning. Patients using CGM exhibited poorer reaction times relative to their healthy peers, despite the benefits afforded by these devices. This may reflect the cognitive load imposed by constant vigilance, device alarms, and the psychological stress associated with real-time awareness of glycemic fluctuations (13). These findings align with the concept of *alarm fatigue* (45), wherein the continuous stream of glucose data and repeated alerts may place sustained demands on attentional and executive control systems. Over time, this persistent cognitive strain—combined with the need for rapid micro-decisions—can lead to attentional fatigue and diminished cognitive efficiency (35). Conversely, users of SMBG, who rely on intermittent finger-prick testing and receive less frequent feedback, exhibited higher levels of hyperactivity and impulsivity relative to controls. This may stem from behavioral dysregulation caused by uncertainty between glucose checks and a lack of continuous glycemic feedback. Moreover, the physical discomfort and stress associated with repeated finger pricks may contribute to an increased cognitive burden (46). Prior studies have also linked hyperglycemia in SMBG users to externalizing behaviors, possibly reflecting emotional and behavioral responses to fragmented or inconsistent glycemic information (47). Our results suggest that insulin therapy modality may impact cognitive functioning in pediatric T1D. Although no overall differences emerged between CSII and MDI users, *post hoc* analysis showed that children using CSII had significantly poorer sustained attention (p = 0.007) compared to controls. This may reflect greater cognitive demands from diabetes self-management or higher disease severity. The MDI group exhibited significantly slower reaction times (p = 0.045) and higher levels of hyperactivity (p = 0.024) relative to controls. These findings may suggest that traditional insulin injection regimens—typically requiring more structured routines and frequent decision-making—could be associated with elevated cognitive and behavioral strain, particularly in youth managing diabetes without technological support (48). Moreover, the difference in reaction time between the CSII group and controls approached significance (p = 0.066), suggesting a potential trend that warrants further exploration.

These outcomes are consistent with prior studies indicating that cognitive profiles in pediatric T1D may be shaped not only by glycemic control but also by the specific treatment modality, as different regimens place varying demands on attentional and executive systems (23, 49). Future research is warranted to fully understand these nuanced associations and should investigate the interactions between insulin delivery methods, diabetes self-management behaviors, and neurocognitive outcomes, using longitudinal designs and real-time glucose monitoring.

### 4.4 Clinical implications and future directions

The cognitive challenges observed in children and adolescents with T1D-particularly in sustained attention, reaction time, and hyperactivity-highlight the need for integrative, multidisciplinary care approaches. Cognitive training programs targeting executive functions and attentional control may enhance self-management capabilities, while psychological interventions aimed at stress reduction and resilience building may mitigate the emotional burden associated with chronic disease management (50).

Our findings support the inclusion of routine neuropsychological screening in pediatric diabetes care. Periodic assessments of cognitive and emotional functioning could facilitate early identification of at-risk individuals and enable timely intervention. Given the association between metabolic control (HbA1c) and cognitive outcomes, optimizing glycemic stability remains a critical therapeutic target. Additionally, combining psychophysiological sensors with computerized neuropsychological testing may offer novel insights into the dynamic interplay between metabolic status and cognitive performance, informing the development of personalized therapeutic strategies.

### 4.5 Study limitations

This study has several limitations. Its cross-sectional design precludes causal inferences between type 1 diabetes and cognitive functioning. Although pre-test glucose levels were recorded, real-time glycemic variability during MOXO-CPT administration was not monitored, limiting conclusions about acute metabolic effects on attention and reaction time. Future research could integrate CGM to address this. The analysis did not include severe glycemic events (e.g., hypoglycemia, ketoacidosis), although such data were collected and will be explored in future analyses. Additionally, the absence of participants with mid-range HbA1c values (7–8%) limits insights into cognitive differences across the full glycemic spectrum. Lack of socioeconomic data is another limitation, as variables like parental education or income may affect cognitive outcomes. Finally, while the MOXO-CPT offers age- and gender-normed data, complementary tools may be needed to assess other domains such as memory and executive planning.

## 5 Conclusions

Children and adolescents with T1D exhibit deficits in attention, reaction time, and hyperactivity compared to healthy controls. These deficits appear to be influenced by disease presence and, to a lesser extent, by HbA1c, but not by sex or specific treatment modalities (CSII vs. MDI; CGM vs. glucometer). The use of CGM systems or other advanced technologies in diabetes treatment, while beneficial for glycemic control, may also introduce cognitive challenges related to increased attentional demands and the psychological stress associated with these technologies.

Early adolescence emerges as a critical period for impulsivity in T1D patients, reflecting normative neurodevelopmental processes exacerbated by metabolic instability during puberty.

Routine cognitive screening and integrative psychosocial support should be considered essential components of comprehensive diabetes management in pediatric populations. Further research is warranted to elucidate the long-term neurodevelopmental consequences of T1D and to evaluate the efficacy of targeted cognitive interventions.

## Data Availability

The original contributions presented in the study are included in the article/supplementary material. Further inquiries can be directed to the corresponding authors.
